# Modeling variations in the cedi/dollar exchange rate in Ghana: an autoregressive conditional heteroscedastic (ARCH) models

**DOI:** 10.1186/s40064-015-1118-0

**Published:** 2015-07-08

**Authors:** Michael Techie Quaicoe, Frank B K Twenefour, Emmanuel M Baah, Ezekiel N N Nortey

**Affiliations:** Western Royal Montessori School, P. O. Box 860, Takoradi, Ghana; Department of Mathematics and Statistics, Takoradi Polytechnic, P. O. Box 256, Takoradi, Ghana; Department of Statistics, University of Ghana, P. O. Box LG 115, Legon Accra, Ghana

**Keywords:** ARMA, GARCH, ACF, PACF and ARCH effect, Conditional mean with variance, Dollar/cedi exchange rate, Forecasting, Time series models

## Abstract

This research article aimed at modeling the variations in the dollar/cedi exchange rate. It examines the applicability of a range of ARCH/GARCH specifications for modeling volatility of the series. The variants considered include the ARMA, GARCH, IGARCH, EGARCH and M-GARCH specifications. The results show that the series was non stationary which resulted from the presence of a unit root in it. The ARMA (1, 1) was found to be the most suitable model for the conditional mean. From the Box–Ljung test statistics *x-squared* of 1476.338 with p value 0.00217 for squared returns and 16.918 with 0.0153 p values for squared residuals, the null hypothesis of no ARCH effect was rejected at 5% significance level indicating the presence of an ARCH effect in the series. ARMA (1, 1) + GARCH (1, 1) which has all parameters significant was found to be the most suitable model for the conditional mean with conditional variance, thus showing adequacy in describing the conditional mean with variance of the return series at 5% significant level. A 24 months forecast for the mean actual exchange rates and mean returns from January, 2013 to December, 2014 made also showed that the fitted model is appropriate for the data and a depreciating trend of the cedi against the dollar for forecasted period respectively.

## Background

Time series models play an important role in the financial market by describing the underlying structure of an economics variable. With available data for financial market analyses in recent times, there has been an increase in the studies concerning persistent shocks both in the mean as well as the variance of the returns of financial instruments in the market. Many time series especially those occurring in natural sciences and engineering cannot be modeled by linear processes. These kinds of time series can have trends which can be modeled by nonlinear processes.

The particular type of non-linear model that is used in finance is known as the autoregressive conditional heteroscedastic—ARCH model (Engle 1982). In the application of financial time series where the variance of the error term is very unlikely to be constant over time, ARCH models are used to describe the behavior of the volatility structure of the error term. They are employed commonly in modeling the volatility structure of financial data and financial indices in order to identify similarities and differences in the structure of the variance of the error term of the observed series.

The basic underlying assumption of the least squares model is that the expected value of all error terms, when squared, is the same at any given point. This assumption is called homoscedasticity (Engle 2001). In volatility analysis however, the variance of the residuals depends on past history and we face heteroscedasticity because the variance is changing over time. A basic means of dealing with heteroscedasticity is to have the variance depending on the lagged period of the squared error terms. ARCH models allows the conditional variance to be dependent upon its own previous lags. In the GARCH (p, q) model (Bollerslev 1986), the conditional variance is dependent upon q lags of the squared error term and p lags of the conditional variance which is very effective to capture the volatility nature of data in financial time series. ARCH and GARCH models treat heteroscedasticity as a variance to be modeled and are most often used in financial theory and practice.

In this article, the authors’ main idea is to use the ARCH/GARCH Specification for modeling the volatility structure of the monthly exchange rate of the Cedi and the US dollar by explaining the volatility structure of the residuals obtained under the best suited mean model for the observed series. This study is significant since the exchange rate of a currency is essential in determining: the level of imports and exports as companies/institutions that rely on import/export can estimate the cost of these import/export with respect to variations in the exchange rate; the country’s level of business activities, Gross Domestic Product (GDP) and employment level and the purchasing power of a local currency i.e. to see whether the currency is appreciating or depreciating against other foreign currencies.

## Analysis of results and discussion

### Exchange rate distribution

**Figure 1:**
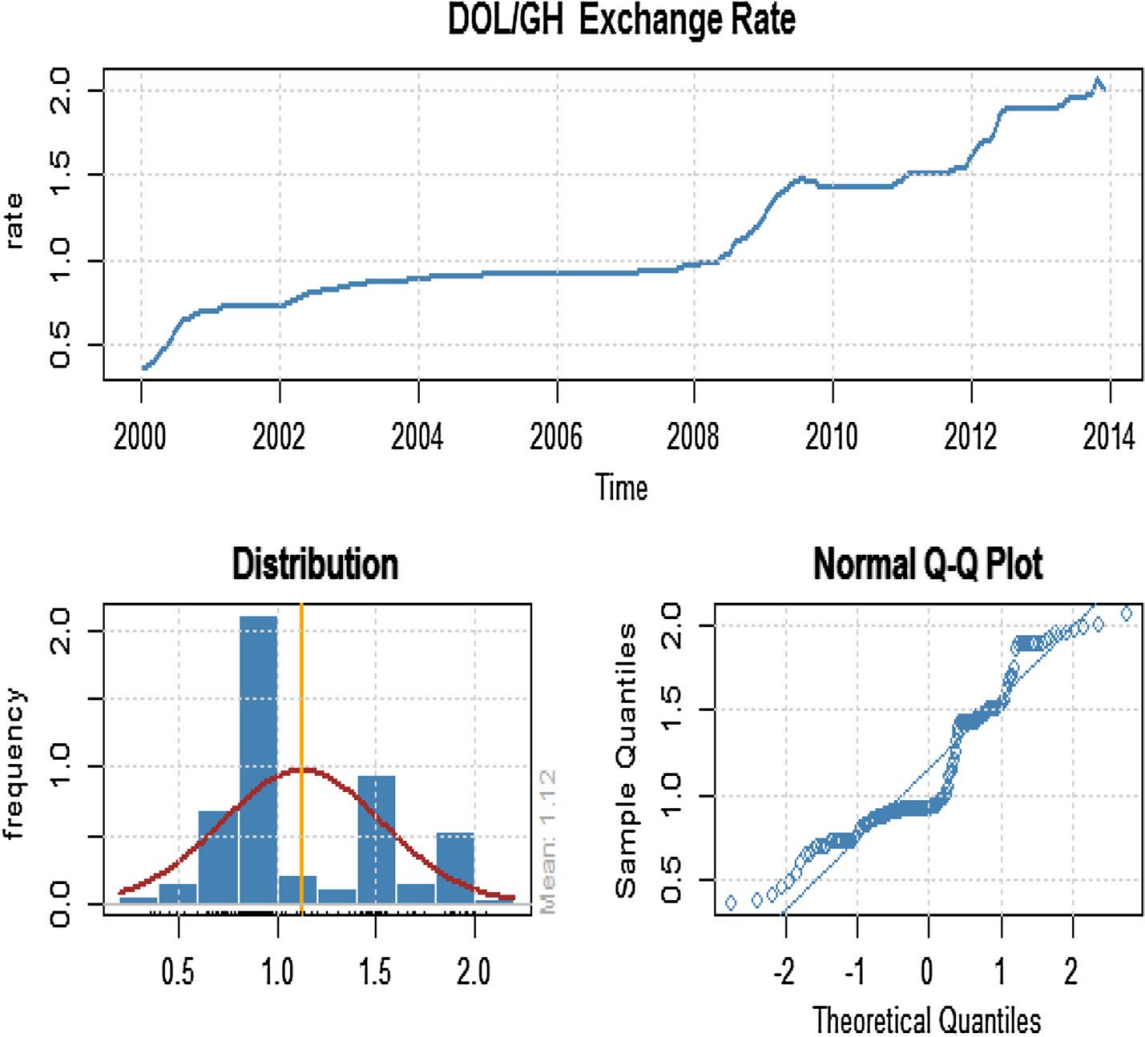
Time plot of monthly exchange rate, distribution and normal Q–Q plot for the dollar/cedi from January, 2000 to December, 2013.

**Figure 2:**
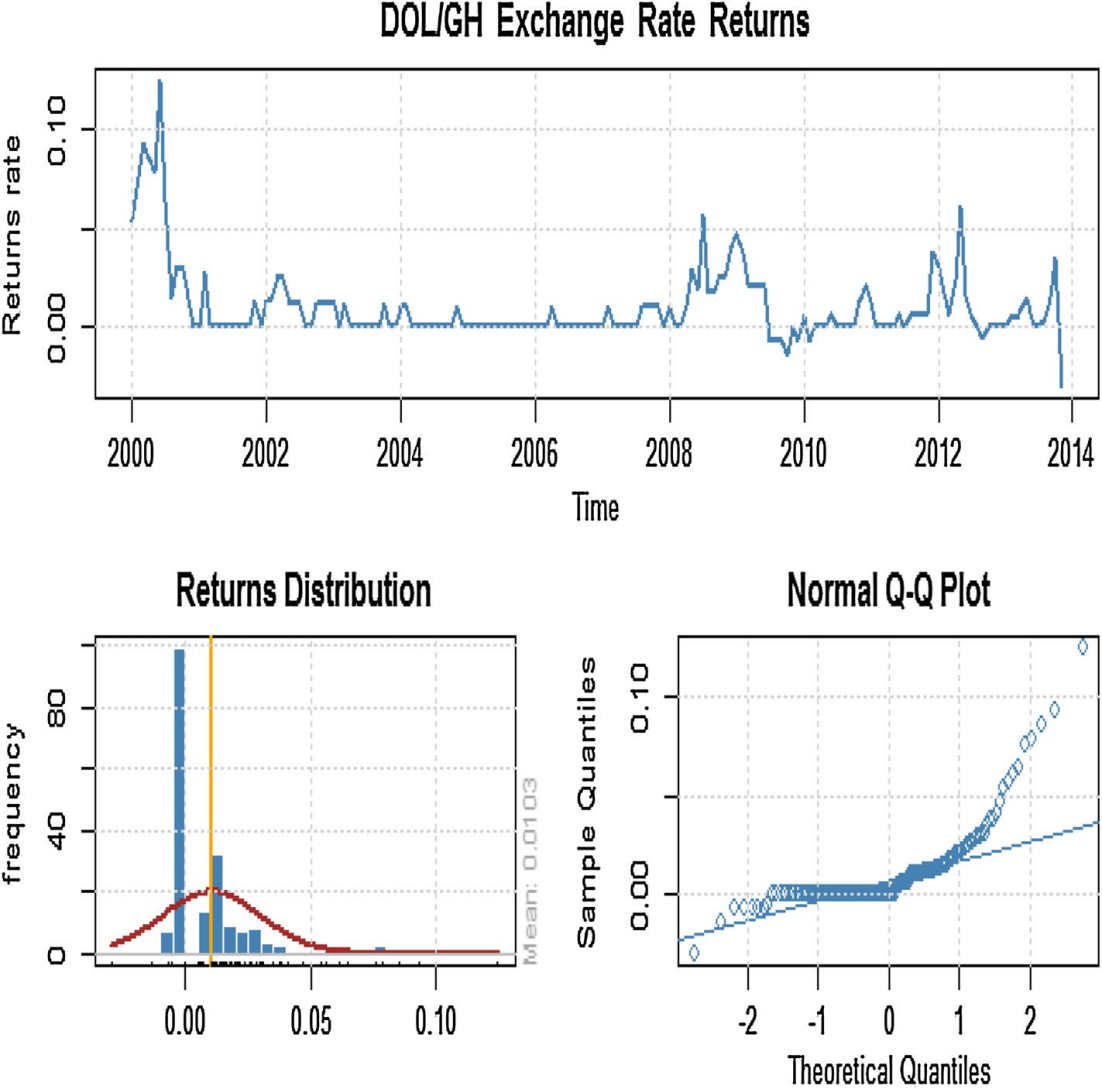
First difference of rate return series.

It was observed in Figure 1 that mean of exchange rate changes over time, which suggests that the series is non-stationary. By performing the unit root test on the series, it was observed that the ADF test statistic (−*2.3232*) is higher than the critical value at a *5%* significance level (−*2.86431*), indicating that we fail to reject the null hypothesis that there is a unit root in the series which is supported by a p value of 0.4414. In order to eliminate the unit root, we found the first differences in *In*(*Rate*), thus $return\,\, = \,\,In\,(Rate)_{t} - \,\,In\,(Rate)_{t - 1}$, and did the test again. ADF test statistic for the rate return is (−*8.0057*), with a p value of *0.01* which indicate that we now reject the null hypothesis of unit root in the series. Hence we conclude that the rate return series is stationary (see Figure 2).

### Determining order of dependency of returns series

**Figure 3:**
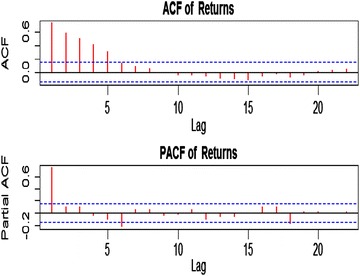
ACF and PACF of returns.

From Figure 3, the autocorrelation and partial autocorrelation functions showed dependency in the return series which required correlation structure in conditional mean. It can also be observed that the model for the conditional mean is ARMA (1, 1) which is given by:

$$r_{t} = 0.999851r_{t - 1} + \,\,0.518176\,\varepsilon_{t - 1} + \varepsilon_{t} ,$$

as shown in Table 1.

**Table 1:** ARMA (1, 1) model parameter estimates

Variable	Coefficient	Standard error	T-statistics	Probability
AR (1)	0.999851	0.004673	213.985	2.00E−16
MA (1)	0.518176	0.077013	6.728	1.72E−11

Source: result from analysis of data, 2014. $\sigma^{2}$ = 0.0002664, conditional sum of squares = 0.04, AIC = −899.97.

### Test for ARCH effect

**Figure 4:**
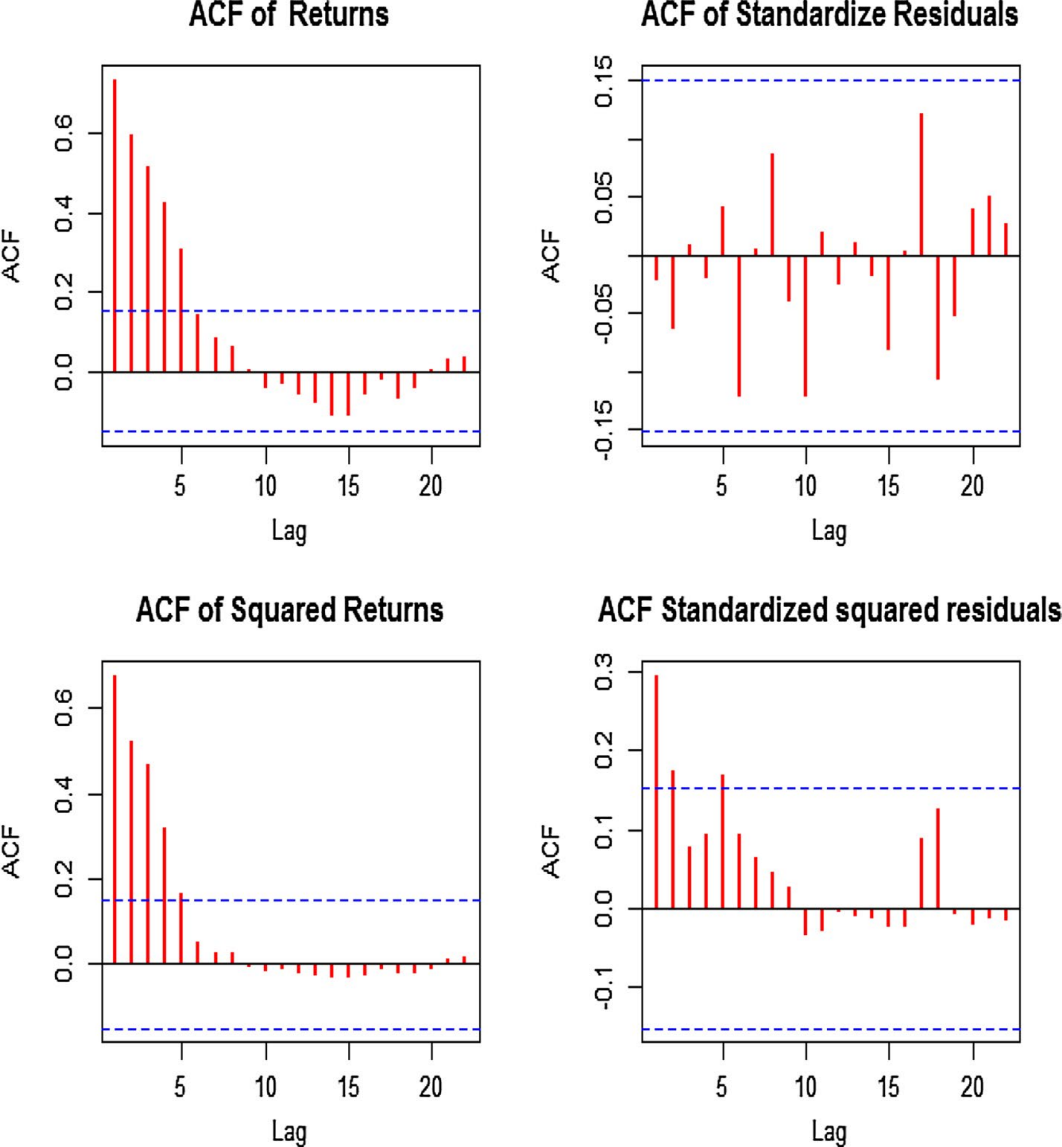
ACF and PACF of the squared residuals and squared returns.

We continue the analysis with a test for an ARCH effect present in the specified model ARMA (1, 1). We first looked at the ACFs of the squared residual and squared returns. Figure 4 presents the AFC of the squared residuals of the fitted model and squared returns respectively. The ACF showed dependency in both the squared residuals and squared returns. We notice that the residuals are not normally distributed which suggest the presence of ARCH effect in the series. This is confirmed by the Box–Ljung test statistics, *1476.338* with *0.000* p value for the squared returns and *16.9183* and a p value of *0.00153* for the squared residuals. Hence the null hypothesis of no ARCH effect is rejected and concluded that there is an ARCH effect in the series.

To eliminate the ARCH effect, GARCH (1, 1) was found to be the most suitable model for the conditional variance, with standardized residual test showing no serial correlation in standardized squared residual at different lags indicating that it is adequate in describing the dynamic volatility of the return series.

From Table 2, the assumed conditional volatility model for the return series is given by;

$$r_{t} = 0.001923 + \varepsilon_{t}$$

$$\varepsilon_{t} \approx N(0,\sigma_{t}^{2} )$$

$$\sigma_{t}^{2} = w_{t} + \alpha_{1} \varepsilon_{t - 1}^{2} + \beta_{1} \sigma_{t - 1}^{2}$$

**Table 2:** GARCH (1, 1) model’s parameter estimates

Variable	Coefficients	Standard error	T-statistic	Probability
Mean	0.001923	0.0004442	4.329	0.000015
Omega	0.00001427	0.000004939	2.89	0.003843
Alpha	1	0.2271	4.403	0.0000107
Beta	0.2711	0.02162	3.785	0.000154

Source: result from analysis of data, 2014.

The variance equation is given by

$$\sigma_{t}^{2} = 0.00001427 + \varepsilon_{t - 1}^{2} + 0.2711\sigma_{t - 1}^{2}$$

### Standardized residual test for GARCH (1, 1)

The Jarque–Bera test for normality in Table 3 was *152.9664* and the ARCH LM of *4.663342* with *pvalue* <*0.001* and *0.96829* respectively which shows that there is no ARCH effect in the standardized squared residuals. The Ljung–Box statistics of standardized residuals for autocorrelation for lags 10, 15 and 20 are *23.50554*, *27.92452* and *31.84835* with p value of *0.00903*, *0.02205* and *0.04494* respectively. Standardized squared residuals at lags 10, 15 and 20 are *5.1266332*, *9.760762* and *11.83056* with respective p value of *0.88256*, *0.83451* and *0.92178*. These values showed that there is no serial correlation in standardized squared residual indicating that the model is adequate in describing the dynamic volatility of the return series.

**Table 3:** Standardized residual test of GARCH (1, 1)

Residual test	Variable	Test statistic	Test value	Probability
Jarque–Bera	*R*	$\chi^{2}$	152.9664	0
Shapiro–Wilk	*R*	W	0.8465621	5.965E−12
Ljung–Box	*R*	$Q(10)$	23.50554	0.009026764
Ljung–Box	*R*	$Q(15)$	27.92452	0.022046764
Ljung–Box	*R*	$Q(20)$	31.84835	0.0449413
Ljung–Box	*R*	$Q(10)$	5.126632	0.8825598
Ljung–Box	*R*	$Q(15)$	9.760762	0.8345127
Ljung–Box	*R*	$Q(20)$	11.83056	0.9217843
LM Arch	*R*	$TR^{2}$	4.663342	0.9682894

Source: result from analysis of data, 2014.

### Model diagnostics of GARCH (1, 1)

**Figure 5:**
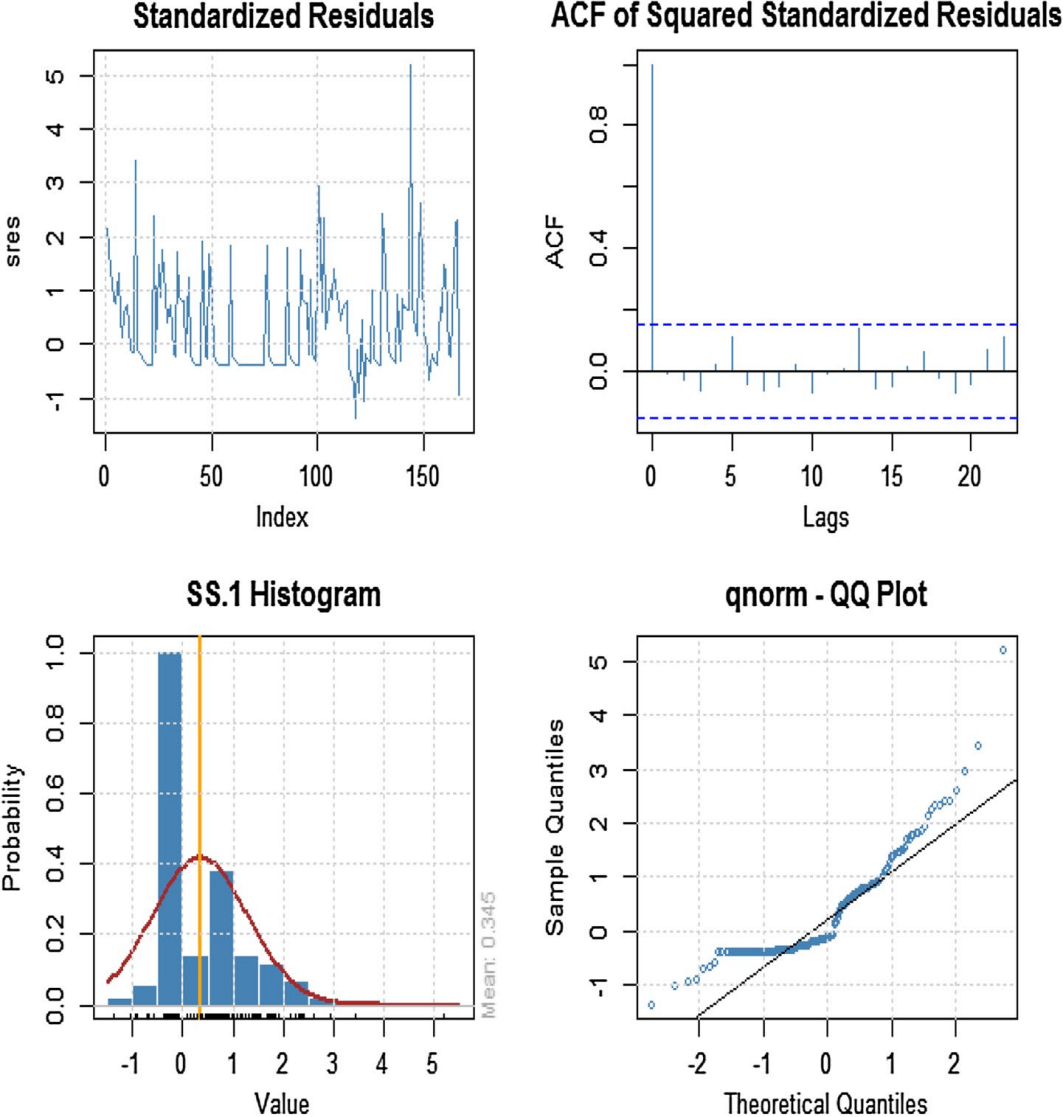
Time plot of standardized residuals, ACF and distribution of standard residual.

The time plot of the standardized residuals in Figure 5 shows no obvious patterns but we notice a spike around the 148th observation. The ACF of the standardized residuals and squared standardized residuals also show no apparent departure from the model assumptions. The histogram and generalized q-norm q–q plot of the standardized residuals show no departure from model assumptions (i.e. the assumed conditional distribution captured the high kurtosis and the heavy tails of the residuals). This suggests the residuals are independent generalized error distribution hence the model is adequate to describe the changing volatility of the returns.

### Returns and variance equation

The conditional mean with conditional variance equation is given by

$$r_{t} = 0.8097r_{t - 1} - 0.5749\varepsilon_{t - 1} + \varepsilon_{t} + 0.00002507\varepsilon_{t - 1}^{2} + 0.1984\sigma_{t - 1}^{2}$$

(see Table 4).

**Table 4:** ARMA (1, 1) + GARCH (1, 1) model’s parameter estimates

Variable	Coefficients	Standard error	T-statistic	Probability
AR (1)	0.8097	0.03542	22.862	2.00E−16
MA (1)	−0.5749	0.107	−5.194	2.06E−07
Omega	0.00002507	0.000006437	3.894	9.85E−05
Alpha	1.00000	0.12798	3.574	0.000352
Beta 1	0.1984	0.09501	2.088	0.03688

Source: result from analysis of data, 2014.

### Standardized residual test of ARMA (1, 1) + GARCH (1, 1)

The Jarque–Bera test for normality was *170.0431* and the ARCH LM of *9.613653* with p value <*0.001* and *0.6498134* respectively which shows that there is no ARCH effect in the standardized squared residuals. The Ljung–Box statistics of standardized residuals for autocorrelation for lags 10, 15 and 20 are *8.937746*, *16.65662* and *20.21719* with p value of *0.5380217*, *0.3398028* and 0*.4444191* respectively. Standardized squared residuals at lags 10, 15 and 20 are *6.394848*, *8.093766* and *10.61964* with respective p-value of *0.7810711*, 0*.9199685* and *0.9554965*. These showed that there is no serial correlation standardized squared residual indicating that the model is adequate in describing the dynamic volatility of the return series as shown in Tables 5, 6.

**Table 5:** Standardized residual test of ARMA (1, 1) + GARCH (1, 1)

Residual test	Variable	Test statistic	Test value	Probability
Jarque–Bera	*R*	$\chi^{2}$	170.0431	0
Shapiro–Wilk	*R*	*W*	0.8750428	1.368657E−10
Ljung–Box	*R*	$Q(10)$	8.937746	0.5380217
Ljung–Box	*R*	$Q(15)$	16.65662	0.3398028
Ljung–Box	*R*	$Q(20)$	20.21719	0.4444191
Ljung–Box	*R* ^2^	$Q(10)$	6.394848	0.7810711
Ljung–Box	*R* ^2^	$Q(15)$	8.093766	0.9199685
Ljung–Box	*R* ^2^	$Q(20)$	10.61964	0.9554965
LM ARCH	*R*	$TR^{2}$	9.613653	0.6498134

Source: result from analysis of data, 2014.

**Table 6:** Information criteria statistics of ARMA (1,1) +GARCH (1,1)

AIC	BIC	SIC	HQIC
−6.319293	−6.225940	−6.321017	−6.281403

Source: result from analysis of data, 2014.

### Model validation

Model validation was conducted to check the validity of the findings made from the analysis. ARMA (1, 1) with GARCH (1, 1) variance model for the return was used to predict the last 12 and next 12 observations by constructing a model each with one-step-ahead prediction of the next observations. The fitted model was used to predict mean actual exchange rates for the next 2 years. That is data up to December, 2012 were used to predict the mean actual rates for 2013 and up to December, 2013 for 2014 mean exchange rates respectively. It can be observed from Table 7 that the mean exchange rates forecasted are very close to the mean actual rates for the forecasted period suggesting that the fitted model is appropriated for the data.

**Table 7:** Mean forecast of actual exchange rates for 2013/2014

Year (2013)	Actual rates	Forecasted rates	Year (2014)	Actual rates	Forecasted rates
January	1.88	1.89	January	2.40	2.00
February	1.88	1.90	February	2.52	2.00
March	1.89	1.90	March	2.68	2.00
April	1.90	1.90	April	2.80	2.00
May	1.92	1.90	May	2.90	2.00
June	1.95	1.95	June	3.00	2.00
July	1.95	1.95	July	3.03	2.00
August	1.95	1.98	August	–	2.10
September	1.96	1.98	September	–	2.35
October	1.99	1.99	October	–	3.00
November	2.06	2.01	November	–	3.08
December	2.00	1.99	December	–	3.42

### Model diagnostic of conditional returns with conditional variance

**Figure 6:**
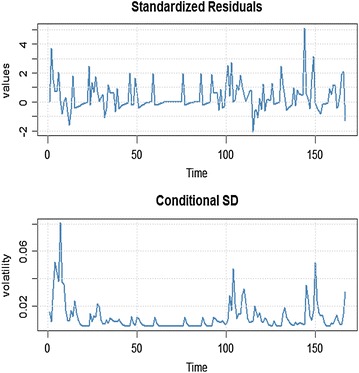
Conditional standard deviation and standardized residuals of ARMA (1, 1) + GARCH (1, 1).

**Figure 7:**
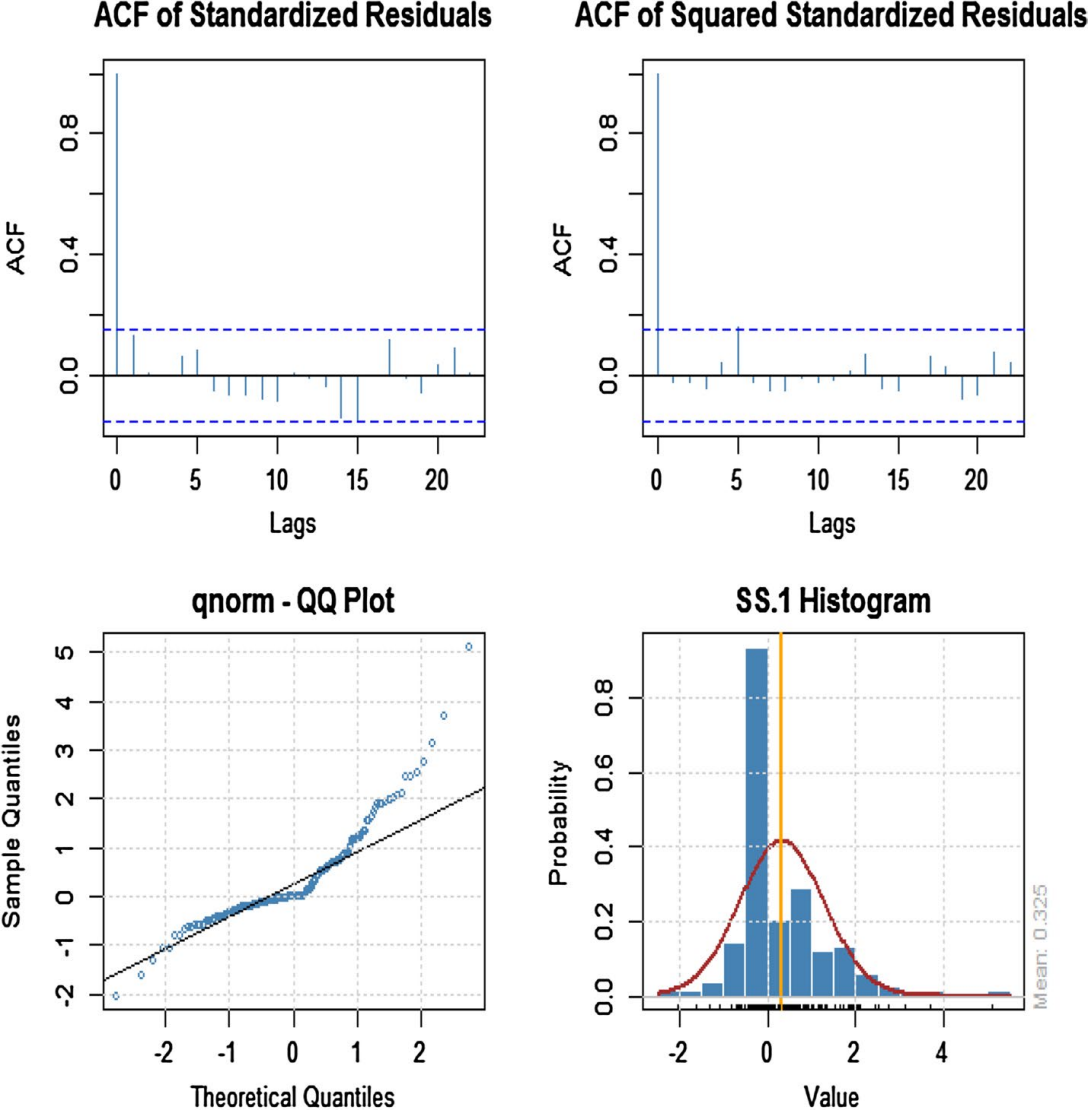
Standardized residual distribution of ARMA (1, 1) + GARCH (1, 1).

The time plot of the standardized residuals in Figure 5 shows no obvious patterns but we notice a spike around the 148th observation. The ACF of the standardized residuals and squared standardized residuals show no apparent departure from the model assumptions as shown in Figure 6. The histogram and generalized q-norm q–q plot of the standardized residuals in Figure 7 show no departure from model assumptions (i.e. the assumed conditional distribution captured the high kurtosis and the heavy tails of the residuals). This suggests the residuals are independent generalized error distribution hence the model seems to be adequate for the data.

### Prediction of next 24 observations of mean returns

**Figure 8:**
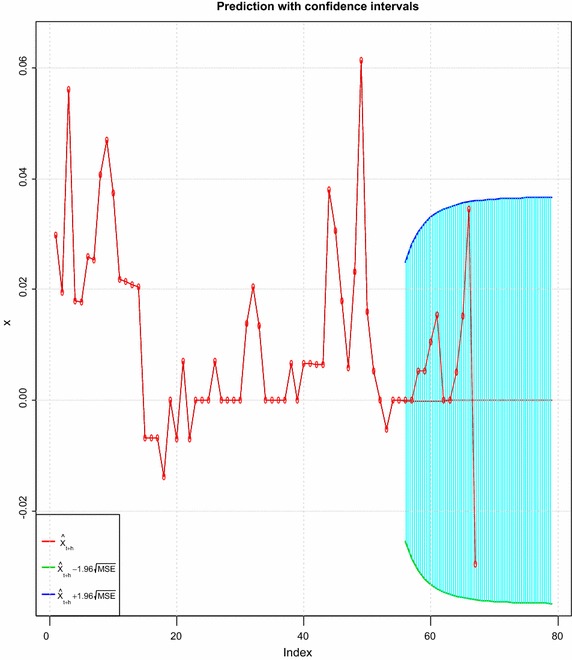
Time plot of forecasted mean returns.

The fitted model was again employed to predict the mean returns for the next 2 years. That is data from January, 2000 to December, 2013 was used to forecast 2014/2015 mean returns. The time plot for the forecasted mean returns is shown in Figure 8. It was observed that the actual mean returns and the predicted mean returns values for the forecasted period lies within the 95% confidence intervals, indicating that the model fitted is adequate in describing the volatility nature of the observed series. The negative values obtained for the mean forecast of returns shows that the Ghana cedi is going to depreciate against the US dollar for the whole period forecasted (see Table 8).

**Table 8:** Mean forecast of returns for 2014/2015

Mean forecast	Mean error	Stand. dev.	95% confidence interval	
			Lower limit	Lower limit
−0.00036229	0.00545061	0.00545061	−0.01032071	0.01104529
−0.00030768	0.0066149	0.00646497	−0.0126573	0.01327265
−0.0002613	0.00753796	0.00725586	−0.01451283	0.01503543
−0.00022191	0.00829912	0.00789875	−0.01604408	0.01648789
−0.00018845	0.00894054	0.0084343	−0.01733467	0.01771158
−0.00016005	0.00948834	0.00888767	−0.01843694	0.01875703
−0.00013592	0.00996082	0.00927586	−0.01938693	0.01965877
−0.00011343	0.01037087	0.00961104	−0.0202111	0.02044196
−0.00009803	0.01072863	0.0099023	−0.02092969	0.02112575
−0.00008325	0.01104203	0.01015669	−0.02155874	0.02172524
−0.0000707	0.01131748	0.01037977	−0.02211116	0.02225256
−0.00006004	0.01156023	0.01057605	−0.02259758	0.02271767
−0.00005099	0.01177463	0.01074922	−0.02302686	0.02312885
−0.00004331	0.01196438	0.01090236	−0.02340644	0.02349305
−0.00003678	0.01213257	0.01103803	−0.02374262	0.02381618
−0.00003423	0.01228188	0.01115844	−0.02404081	0.02410327
−0.00002653	0.01241459	0.01126544	−0.02430562	0.02435867
−0.00002253	0.01253267	0.01136065	−0.02454106	0.02458611
−0.00001913	0.01263785	0.01144546	−0.02475059	0.02478885
−0.00001625	0.0127316	0.01152107	−0.02493724	0.02496973
−0.0000138	0.01281524	0.01158854	−0.02510362	0.02513121
−0.00001172	0.01288991	0.01164878	−0.02525204	0.02527547
−0.00000995	0.0129566	0.0117026	−0.02538453	0.02540443
−0.00000845	0.01301621	0.0117507	−0.02550285	0.02551976

Source: result from analysis of data, 2014.

## Conclusions

The observed series does not change over time, thus showing that the series is stationary; hence the probability law that governs the behavior of the process does not change over time. The distribution of the return series is not normal with non-constant variance skewed to the right. The model that explains the stochastic mechanism of the observed series is ARMA (1, 1) + GARCH (1, 1). That is the optimum model for the dollar/cedi exchange rate returns (conditional mean with non-constant variance). The time series components found in the model were trend and random variation. It was also observed that, the ARMA (1, 1) + GARCH (1, 1) fitted is adequate for treating the series’ heteroscedasticity by modeling the variations in the series which is the main objective of this study. The forecasts were found to have upward trend for the 2 years (2014/2015) period, indicating that the cedi will continue to depreciate against the dollar for the forecasted period.

## Recommendations

Based on the findings, it is recommended that; there should be an increase in the supply of foreign currencies into the local market and absorbing some of the excess liquidity from the economy. The government should consider the volatility of other macroeconomic variables in making both policy and investment decision. The fiscal and monetary policies adopted should be revised to help address the depreciation of the cedi.

## Methods

This paper employs a model based on information and real data obtained from the Bank of Ghana and the Ghana Stock Exchange. The samples include monthly data excluding weekends and holidays, of the average nominal exchange rate of the Ghana cedi/the US dollar, for the period January, 2000 to December 2013, comprising 168 data points. ARCH type models of Time Series Analysis and the statistical computing package R were used for the modeling. The stationarity of data is usually described by the time plots and the correlogram. The unit root test determines whether a time series is stable around its level or stable around the difference in its level (Dickey–Fuller or the Augmented Dickey–Fuller root test).

### ARMA (p, q) model

The general ARMA (p, q) model is given by

1 $$r_{t} = \phi_{o} + \,\sum\limits_{i = 1}^{p} {\phi_{i} r_{t - 1} + \,a_{t} - \sum\limits_{i = 1}^{q} {\phi_{i} a_{t - 1} } }$$

where $a_{t}$ a white noise series and p and q are nonnegative integers. The Autoregressive (AR) and Moving average (MA) models are special cases of the ARMA (p, q) model. Using the back-shift operator, the model can be written as

2 $$\begin{aligned} &(1 - \phi_{1} \beta - \cdots - \phi_{p} \beta^{p} )r_{t} \hfill \\ &= \phi_{o} + (1 - \theta_{1} \beta \cdots - \theta \beta^{q} )a \hfill \\ \end{aligned}$$

The polynomial $1 - \phi_{1} \beta - \cdots - \phi_{p} \beta^{p}$ is the AR polynomial of the model. Similarly, $1 - \theta_{1} \beta \cdots - \theta \beta^{q}$ is the MA polynomial. It is required that there are no common factors between the AR and MA polynomials; otherwise the order of the model can be reduced (Noh, Engle and Kane 1994; DeLurgio 1998; Keller and Warrack 2003).

### ARCH (1) model

The first and simplest heteroscedastic model we will look at is the ARCH model, which stands for Autoregressive Conditional Heteroscedasticity. The AR comes from the fact that these models are autoregressive models in squared returns. The conditional comes from the fact that in these models, next period’s volatility is conditional on information this period. Heteroscedasticity means non constant volatility. In a standard linear regression where $y_{i} = \alpha + \beta x_{i} + \varepsilon_{i}$, when the variance of the residuals, $\varepsilon_{i}$ is constant, we call that homoscedastic and use ordinary least squares to estimate $\alpha$ and $\beta$. If, on the other hand, the variance of the residuals is not constant, we call that heteroscedastic and we can use weighted least squares to estimate the regression coefficients (Noh et al. 1994; DeLurgio 1998; Keller and Warrack 2003).

Let us assume that the return on an asset is $r_{t} = \mu + \sigma_{t} \varepsilon_{t}$ where $\varepsilon_{t}$ is a sequence of N (0, 1) i.i.d. random variables. We will define the residual return at time t, $r_{t} - \mu$, as $a_{t} = \sigma_{t} \varepsilon_{t} .$ In an ARCH (1) model, first developed by Engle (1982), $\alpha_{t} = \alpha_{0} + \alpha_{1} a_{t - 1}^{2}$, where $\alpha_{0}$ >0 and $\alpha_{1} \ge 0$ to ensure positive variance and 1 < 1 for stationarity.

Under an ARCH (1) model, if the residual return, $a_{t}$ is large in magnitude, our forecast for next period’s conditional volatility, $\sigma_{t + 1}$ will be large. We say that in this model, the returns are conditionally normal (conditional on all information up to time $t - 1$, the one period returns are normally distributed). Also, note that the returns; $r_{t}$ are uncorrelated but are not identically independent distributed (Engle 1982, 2001).

The unconditional variance of $a_{t}$ is;

3 $$\begin{aligned} \text{var} (a_{t} ) &= E\left[ {a_{t}^{2} } \right] - (E\left[ {a_{t} } \right])^{2} \hfill \\ &= E\left[ {a_{t}^{2} } \right] \hfill \\ &= E\left[ {\sigma_{t}^{2} \varepsilon_{t}^{2} } \right] \hfill \\ &= E\left[ {a_{t}^{2} } \right] \hfill \\ &= \alpha_{0} + \alpha_{1} E\left[ {a_{t - 1}^{2} } \right] \hfill \\ \end{aligned}$$

Since $a_{t}$ is a stationary process, the $\text{var} \left( {a_{t} } \right) = \text{var} \left( {a_{t - 1} } \right) = E\left[ {a_{t - 1}^{2} } \right]$, then,

4 $$\text{var} \left( {a_{t} } \right) = \frac{{\alpha_{0} }}{{1 - \alpha_{1} }}$$

An ARCH (1) is like an AR (1) model on squared residuals $a_{t}^{2}$. To see this, we define the conditional forecast error, or the difference between the squared residual return and our conditional expectation of the squared residual return is given as;

5 $$\begin{aligned} v_{t} &= a_{t}^{2} - E \left\langle {a_{t}^{2} } || {I_{t - 1} } \right\rangle \\ v_{t} &= a_{t}^{2} - \sigma_{t}^{2} \hfill \\ \end{aligned}$$

where $I_{t - 1}$ is the information at time $t - 1$, note that $v_{t}$ is a zero mean, uncorrelated series. The ARCH (1) equation becomes

6 $$\begin{aligned} \sigma_{t}^{2} &= \alpha_{0} + \alpha_{1} a_{t - 1}^{2} \hfill \\ a_{t}^{2} - v_{t} &= \alpha_{0} + \alpha_{1} a_{t - 1}^{2} \hfill \\ a_{t}^{2} &= \alpha_{0} + \alpha_{1} a_{t - 1}^{2} + v_{t} \hfill \\ \end{aligned}$$

This is an AR (1) process on squared residuals.

### GARCH (1, 1) model

In an ARCH (1) model, next period’s variance only depends on last period’s squared residual so a crisis that caused a large residual would not have the sort of persistence that we observe after actual crises. This has led to an extension of the ARCH model to a GARCH, or Generalized ARCH model, first developed by Bollerslev (1986), which is similar in spirit to an ARMA model (Noh et al. 1994; DeLurgio 1998; Keller and Warrack 2003).

$$\begin{aligned} \sigma_{t}^{2} = \alpha_{0} + \alpha_{1} a_{t - 1}^{2} + \beta_{1} \sigma_{t - 1}^{2} \hfill \\ \hfill \\ \end{aligned}$$

where $\alpha_{0}$ >0, $\alpha_{1}$ >0, $\beta_{1}$ >0, and $\alpha_{1} + \beta_{1}$ <1, so that our next period forecast of variance is a blend of our last period forecast and last period’s squared return. We can see that just as an ARCH (1) model is an AR (1) model on squared residuals, an ARCH (1, 1) model is an ARMA (1, 1) model on squared residuals by making the same substitutions as before $\varvec{v}_{\varvec{t}} = a_{t}^{2} - \sigma_{t}^{2}$.

$$\begin{aligned} \sigma_{t}^{2} &= \alpha_{0} + \alpha_{1} a_{t - 1}^{2} + \beta_{1} \sigma_{t - 1}^{2} \hfill \\ a_{t}^{2} - v_{t} &= \alpha_{0} + \alpha_{1} a_{t - 1}^{2} + \beta_{1} \left( {a_{t - 1}^{2} - v_{t - i} } \right) \hfill \\ a_{t}^{2} &= \alpha_{0} + \left( {\alpha_{1} \beta_{1} } \right)a_{t - 1}^{2} + v_{t} - \beta_{1} v_{t - 1} \hfill \\ \end{aligned}$$

This is an ARMA (1, 1) on the squared residuals. The unconditional variance of $a_{t}$ is

7 $$\begin{aligned} \text{var} \left( {a_{t} } \right) &= E\left[ {a_{t}^{2} } \right] - \left( {E\left[ {a_{t} } \right]} \right)^{2} \hfill \\ & = E\left[ {a_{t}^{2} } \right] \hfill \\ & = E\left[ {\sigma_{t}^{2} \varepsilon_{t}^{2} } \right] \hfill \\ & = E\left[ {\sigma_{t}^{2} } \right] \hfill \\ & = \alpha_{0} + \alpha_{1} E\left[ {a_{t - 1}^{2} } \right] + \beta_{1} \sigma_{t - 1}^{2} \hfill \\ & = \alpha_{0} + \left( {\alpha_{1} + \beta_{1} } \right)E\left[ {a_{t - 1}^{2} } \right] \hfill \\ & \hfill \\ \end{aligned}$$

And since $a_{t}$ is a stationary process,

8 $$\begin{aligned} \text{var} \left( {a_{t} } \right) = \frac{{\alpha_{0} }}{{1 - \alpha_{1} - \beta_{1} }} \hfill \\ & \hfill \\ \end{aligned}$$

$a_{t} = \sigma_{t} \varepsilon_{t}$, the unconditional variance of returns, $E\left[ {\sigma_{t}^{2} } \right] = E\left[ {a_{t}^{2} } \right]$.

ARMA (1, 1) can be written as an AR ($\infty$), and GARCH (1, 1) can be written as an AR ($\infty$) which yield the following

9 $$\begin{aligned} a_{t}^{2} &= \alpha_{0} + \alpha_{1} a_{t - 1}^{2} + \beta_{1} \sigma_{t - 1}^{2} \hfill \\ & = \alpha_{0} + \alpha_{1} a_{t - 1}^{2} + \beta_{1} \left( {\alpha_{0} + \alpha_{1} a_{t - 2}^{2} + \beta_{1} \sigma_{t - 2}^{2} } \right) \hfill \\ & = \alpha_{0} + \alpha_{1} a_{t - 1}^{2} + \alpha_{0} \beta_{1} + \alpha_{1} \beta_{1} a_{t - 2}^{2} + \beta_{1}^{2} \sigma_{t - 2}^{2} \hfill \\ & = \alpha_{0} + \alpha_{1} a_{t - 1}^{2} + \alpha_{0} \beta_{1} + \alpha_{1} \beta_{1} a_{t - 2}^{2} + \beta_{1}^{2} \left( {\alpha_{0} + \alpha_{1} a_{t - 3}^{2} + \beta_{1} \sigma_{t - 3}^{2} } \right) \hfill \\ & \vdots \hfill \\ & = \frac{{\alpha_{0} }}{{1 - \beta_{1} }} + \alpha_{1} \sum\limits_{i = 0}^{\infty } {a_{t - 1 - i}^{2} \beta_{1}^{i} } \, \hfill \\ \end{aligned}$$

So that the conditional variance at time t is the weighted sum of past squared residuals and the weights decrease as you go further back in time. Since the unconditional variance of returns is

10 $$E\left[ {\alpha^{2} } \right] = \frac{{\alpha_{0} }}{{\left( {1 - \alpha_{1} - \beta_{1} } \right)}}$$

We can write the GARCH (1, 1) equation yet another way

11 $$\begin{aligned} \sigma_{t}^{2} &= \alpha_{0} + \alpha_{1} a_{t - 1}^{2} + \beta_{1} \sigma_{t - 1}^{2} \hfill \\ & = \left( {1 - \alpha_{1} - \beta_{1} } \right)E\left[ {\sigma^{2} } \right] + \alpha_{1} a_{t - 1}^{2} + \beta_{1} \sigma_{t - 1}^{2} \hfill \\ \end{aligned}$$

In this way, it is easy to see that next period’s conditional variance is a weighted combination of the unconditional variance of returns, $E\left[ {\sigma^{2} } \right]$ last period’s squared residuals $a_{t - 1}^{2}$, and last period’s conditional variance, $\sigma_{t - 1}^{2}$, with weights $\left( {1 - \alpha_{1} - \beta_{1} } \right)$, $\alpha_{1}$, $\beta_{1}$ which sum to one. It is often useful not only to forecast next period’s variance of returns, but also to make an $l$-step ahead forecast, especially if our goal is to price an option with $l$ steps to expiration using our volatility model (Noh et al. 1994; DeLurgio 1998; Keller and Warrack 2003).

### The integrated GARCH model

In the case where $\alpha_{1} + \beta_{1} = 1$, the GARCH (1, 1) model becomes

12 $$\sigma_{t}^{2} = \alpha_{0} + \left( {1 - \beta_{1} } \right)a_{t - 1}^{2} + \beta_{1} \sigma_{t - 1}^{2} .$$

This model, first developed by Engle and Bollerslev (1986), is referred to an Integrated GARCH model, or an IGARCH model. Squared shocks are persistent, so the variance follows a random walk with a drift. Since we generally do not observe a drift in variance, we will assume $\alpha_{0} = 0$. Just as a GARCH model is analogous to an ARMA model, the IGARCH model where the variance process has a unit root is analogous to an ARIMA model (Noh et al. 1994; DeLurgio 1998; Keller and Warrack 2003).

When $\alpha_{1} + \beta_{1} = 1$ and $\alpha_{0} = 0$, the $l - step$ ahead forecast that we derived for a GARCH (1, 1) model becomes

13 $$\begin{aligned} \hat{\sigma }_{t + 1}^{2} &= \alpha_{0} + \left( {\alpha_{1} + \beta_{1} } \right)\hat{\sigma }_{t + l - 1}^{2} \hfill \\ & = \hat{\sigma }_{t + l - 1}^{2} \hfill \\ & = \sigma_{t}^{2} \hfill \\ \end{aligned}$$

Thus the forecast for future variance is the current variance, just as in a random walk. Also, if we now write the model as an ARCH ($\infty$) as we did before with a GARCH (1, 1) model, after repeated substitutions we get

14 $$\begin{aligned} \sigma_{t}^{2} &= \frac{{\alpha_{0} }}{{1 - \beta_{1} }} + \alpha_{1} \sum\limits_{i = 1}^{\infty } {a_{t - 1 - i}^{2} \beta_{1}^{i} } \hfill \\ & = \left( {1 - \beta_{1} } \right)\sum\limits_{i = 1}^{\infty } {a_{t - 1 - i}^{2} \beta_{1}^{i} } \hfill \\ & = \left( {1 - \beta_{1} } \right)\left( {a_{t - 1}^{2} + \beta_{1} a_{t - 2}^{2} + \beta_{1}^{2} a_{t - 3}^{2} + \cdots } \right) \hfill \\ \end{aligned}$$

This is an exponential smoothing of past squared residuals. The “weights” on the squared residuals, The ‘weights’ on the squared residuals, $\left( {1 - \beta_{1} } \right),\beta_{1} \left( {1 - \beta_{1} } \right),\beta_{1}^{2} \left( {1 - \beta_{1} } \right), \ldots$ sum to one, so exponentially weighting can be used as an alternative to historical variance (Noh et al. 1994; DeLurgio 1998; Keller and Warrack 2003).

15 $$\sigma_{t}^{2} = \left( {1/N} \right)\sum\limits_{i = 1}^{N} {\left( {r_{t - 1} - \overline{r} } \right)}^{2}$$

### The GARCH-M model

Another variation of a GARCH model tests whether variance can impact the mean of future returns. These models are referred to as GARCH in the mean or GARCH-M models (Noh et al. 1994; DeLurgio 1998; Keller and Warrack 2003).

A GARCH (1, 1)-M is represented as

16 $$\sigma_{t}^{2} = \alpha_{0} + \alpha_{1} a_{t - 1}^{2} + \beta_{1} \sigma_{t - 1}^{2}$$

17 $$r_{t} = \mu + \sigma_{t} \varepsilon_{t} + \lambda \sigma_{t}^{2}.$$

In some specifications, the volatility, rather than the variance, affects returns a, $r_{t} = \mu + \sigma_{t} \varepsilon_{t} + \lambda \sigma_{t}$.

If $\lambda = 0$, these models imply a serial correlation of returns since variance is serially correlated and the returns depend on the variance. Many studies have tried to determine whether $\lambda$ is significantly different from zero, usually with mixed conclusions.

### The EGARCH model

Another variant on GARCH to account for the asymmetry between up and down movement of volatility of financial data is the Exponential Generalized Autoregressive Conditional Heteroscedastic (EGARCH) model of Nelson (1990). In an EGARCH (1, 1),

18 $$In\left( {\sigma_{t}^{2} } \right) = \alpha_{0} + \frac{{\alpha_{1} a_{t - 1} + \gamma \left| {a_{t - 1} } \right|}}{{\sigma_{t - 1} }} + \beta_{1} In\left( {\sigma_{t - i}^{2} } \right)$$

Here there is an asymmetric effect between positive and negative return. Also to avoid the possibility of a negative variance, the model is an AR (1) on $In\left( {\sigma_{t}^{2} } \right)$ rather than $\sigma_{t}^{2}$.

An alternative representation of an EGARCH model which often found in literature is to write the EGARCH as an AR (1) process on $In\left( {\sigma_{t}^{2} } \right)$ with zero mean, independent identically distributed residuals

$$g(\varepsilon_{t} ),\;In\left( {\sigma_{t}^{2} } \right) = \alpha + \beta \left[ {In\left( {\sigma_{t - 1}^{2} } \right) - \alpha } \right] + g\left( {\varepsilon_{t - 1} } \right)$$

where

$$g\left( {\varepsilon_{t} } \right) = \left( {\theta \varepsilon_{t} + \gamma \left| {\varepsilon_{t} } \right| - E\left[ {\left| {\varepsilon_{t} } \right|} \right]} \right) {\rm and}\,\,{\rm if }$$

$$\varepsilon_{t} \sim N\left( {0,1} \right),E\left[ {\left| {\varepsilon_{t} } \right|} \right] = \sqrt {\frac{2}{\pi }}.$$

19 $$\alpha = E\left[ {In\left( {\sigma_{t}^{2} } \right)} \right]$$

There are many other models that try to capture the asymmetry between up and down on future volatility. For example, a slight variation on a GARCH (1, 1) is $\sigma_{t}^{2} = \alpha_{0} + \alpha_{1} \left( {a_{t - 1} - x} \right)^{2} + \beta_{1} \sigma_{t - 1}^{2}$ so that the next period’s variance is not necessarily minimized when the squared residual are zero.

### ARCH effects

ARCH effects can be tested in pre-estimation or post-estimation analysis. In post-estimation, it test the remaining ARCH effect, thus whether or not the conditional heteroscedasticity has been removed. For the purpose of this research, it is applied on standardized residual of the fitted model which is an L M test for the ARCH effect in the residual. An indication of ARCH effects is that the residuals are uncorrelated, but the squared residuals are correlated. Normality test are used to test the behavior of ARCH effect if the normality can be described by the conditional error distribution. Another way is to inspect the autocorrelation structure of the residual and squared residuals using portmanteau tests. These tests are used for diagnostic checking of fitted time series model (Bollerslev 1986; Engle 1982; DeLurgio 1998).

Estimation of GARCH models is done with the normal distribution. Pre-estimation analysis is performed on the return and squared return, which includes important test applied to the two time series to ensure that conditional volatility modeling is appropriate. The main tests before actually estimating the conditional volatility are Engle’s ARCH test and Portmanteau (Bollerslev 1986; Engle 1982).

### Forecast evaluation methods

The selected model for the time series is used to make prediction into the future. After making forecast and choosing a proxy for actual volatility, next step is to choose statistical loss function to see how close the forecast to their target and compare forecast performance of model. Evaluation of performance of different volatility model is built on statistical loss function present in literature. These are based on moment of forecast error such as root mean squared error (RMSE), mean absolute error (MAE), adjusted mean absolute percentage error (AMAPE). The model would be the one that minimize such a function of the forecast errors. Autoregressive forecasting will be used. Here the observed series depends linearly on its own previous values plus a combination of currents and previous values of a white noise error term. Predictions are made by constructing a model each with one-step-ahead prediction of the next observations (Noh et al. 1994; DeLurgio 1998; Keller and Warrack 2003).
